# Challenges in upright limb occlusion pressure determination with the Delfi PTS: pilot data from two independent cohorts

**DOI:** 10.3389/fspor.2025.1686040

**Published:** 2025-10-07

**Authors:** Nicholas Rolnick, Victor S. de Queiros, Campbell Ruffhead, Sean Richard Zupnik, Michael Sergio, Lucas Kuriawa, Tim Werner

**Affiliations:** ^1^ The Human Performance Mechanic, CUNY Lehman College, New York, NY, United States; ^2^ The BFR PROS, New York, NY, United States; ^3^ Department of Physical Education, State University of Paraíba (UEPB), Campina Grande, Brazil; ^4^ Department of Exercise Science, Salisbury University, Salisbury, MD, United States

**Keywords:** blood flow restriction, arterial occlusion pressure, BFR training, reliability, postural influence, pilot study

## Abstract

**Purpose:**

Blood flow restriction (BFR) exercise prescription relies on accurate determination of limb occlusion pressure (LOP), which is known to vary by body position. While recent guidelines suggest assessing LOP in the same position as the intended exercise, the feasibility of upright LOP assessment remains uncertain.

**Methods:**

This pilot study evaluated the feasibility of seated and standing LOP assessment using the Delfi Personalized Tourniquet System (PTS) under multiple postural and cueing conditions. Two separate and independent cohorts (*n* = 11 each; 18–35 years old) completed three experimental conditions involving either equal weightbearing (with/without visual cueing), seated, or wall-supported standing, with force plates used to monitor weight distribution. LOP was measured on two separate days using manufacturer guidelines. In each condition, three assessments were performed (*n* = 66 measurements per experimental condition).

**Results:**

LOP was unable to be determined in enough participants within and between cohorts to be able to compare reliability of LOP measured between conditions. Therefore, Cochran's *Q* test revealed no significant differences in successful LOP detection across conditions in cohort 1 (*p* = 0.234) but did reveal differences in conditions 1 and 3 (*p* < 0.001) and between 2 and 3 (*p* = 0.001) in cohort 2. Overall, we were successful in measuring LOP in 28.7% of the total attempts (57/198) in Cohort 1 and 34.8% (69/198) of the total attempts in Cohort 2. Seated LOP assessment was most successfully measured (34/66 attempts, 51%).

**Conclusion:**

Seated and standing LOP assessment using the Delfi PTS appears largely unfeasible, regardless of cueing or postural modifications. These findings question the feasibility of implementing seated or standing LOP measurements using the Delfi PTS in research and practice and suggest that future BFR research and application may benefit from supine LOP determination, or from developing dynamic calibration protocols suited for upright exercise.

## Introduction

Blood flow restriction (BFR) exercise is typically performed with low loads or intensities, using a pneumatic cuff placed at the most proximal part of the exercising limb (1). Current guidelines recommend individualizing pressure based on limb occlusion pressure (LOP)—the minimum pressure required to fully occlude arterial inflow (1). Prescribing exercise at a percentage of LOP has become the standard approach to ensure consistent and reproducible stimuli across studies and clinical applications. However, resting LOP is not fixed; it varies with body position—often, though not always, increasing from supine to seated to standing during lower-body assessments (2–4). This variability has prompted recommendations to measure LOP in the same position in which exercise is performed as it may impact the BFR stimulus (5). Despite this, evidence directly supporting the acute or long-term relevance of this practice remains limited.

Recent survey data indicate that the Delfi Personalized Tourniquet System (PTS) – an automated and empirically validated BFR cuff system (6) - is among the most widely used BFR devices in practice (7, 8). The Delfi PTS automatically determines LOP with accuracy equivalent to Doppler ultrasound in supine, eliminating the need for user input (6). Consequently, if position-specific LOP measurement is adopted, upright assessments would become standard practice for seated and standing-based BFR exercises. Yet, beyond a single study (5) comparing similar percentages of resting supine and seated LOPs during bilateral leg extension, no research has examined exercise responses when BFR is applied using LOP measured in the seated or standing position compared with a more regressive position (e.g., supine). Although preliminary findings suggest seated and standing resting LOP assessments can be reliable (3), practical challenges remain. Gravitational forces acting on the cuff, involuntary muscle contractions required to maintain upright posture, and the higher pressures typically observed in upright postures make these assessments difficult to execute and reproduce consistently. To the authors knowledge, no automated devices have been validated against doppler ultrasound in seated or standing, possibly due to these practical challenges. Given Delfi PTS's widespread clinical use and capacity to inflate to 350 mmHg, clarifying the feasibility of upright postural LOP assessment is essential to bridge the gap between theoretical recommendations and practical implementation in applied BFR settings.

Accordingly, establishing a reliable and standardized protocol for seated and standing LOP assessment is essential before conducting formal trials that investigate the influence of body position on LOP, as well as its associated physiological and perceptual responses and long-term adaptations during upright exercise contexts such as leg presses, squats, deadlifts, lunges, and standing calf raises using the Delfi PTS. The purpose of this pilot study was twofold. First, we sought to evaluate whether standing LOP can be reliably measured with the Delfi PTS under three conditions: 1) without standardization, 2) with visual cueing for equal weightbearing, and 3) without visual cueing. Second, we aimed to examine whether reliability improves when participants are 1) seated (e.g., the regressive position to standing), 2) lean against a wall to partially support their body weight in the medial-lateral and 3) anterior-posterior directions, again both with visual cueing. We hypothesized that standard standing assessments with equal weight-bearing would be difficult to perform consistently and yield limited usable data, whereas the seated and leaning protocols would demonstrate greater reliability and feasibility for future research requiring standing LOP assessment.

## Materials and methods

### Participants

To address the stated aims, a power analysis was conducted using G*Power (version 3.1.9.7; Kiel University, Germany). The analysis estimated a required sample size of 11 participants for a repeated measures analysis of variance (ANOVA) at *α* = 0.05 and *β* = 0.80 to detect a partial eta-squared (*η*^2^p) of 0.14 (large effect) for detecting differences in occlusion pressures between the three conditions. Accounting for an anticipated 20% attrition rate, 25 participants were recruited. The age range of participants was 18–35 years with diverse racial and ethnic backgrounds. Inclusion criteria were 18–40 years of age, stable body weight (±2.5 kg within the previous six months), and eumenorrhea for at least the last two years in female participants. Exclusion criteria included a diagnosis of diabetes, cardiovascular, liver, and/or kidney disease (9); stage 2 hypertension as defined by the American Heart Association (10); sleep apnea; morbid obesity; acute surgery within two months of data collection; or current/past use of tobacco products within the last five years. All participants provided written informed consent in accordance with the Declaration of Helsinki. The study was approved by the Institutional Review Board of Salisbury University (protocol #442) and registered at clinicaltrials.gov (NCT06718582).

### Experimental design

**Figure 1:**
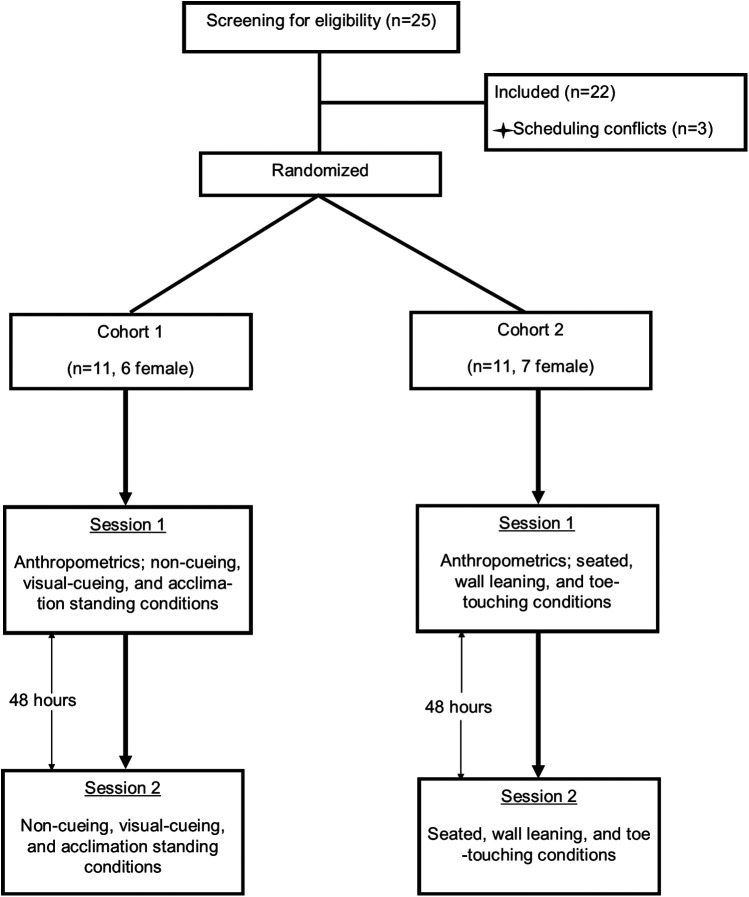
Schematic of study design.

**Figure 2:**
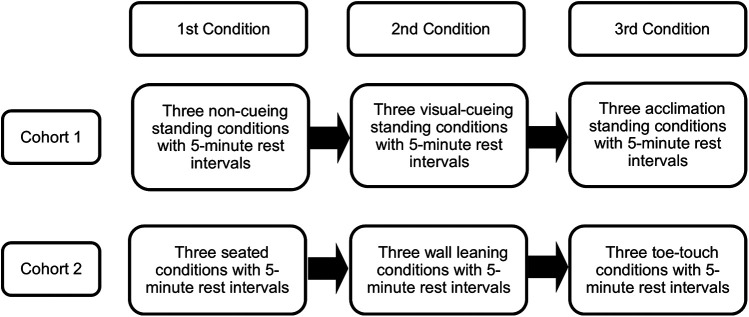
Treatment order for cohort 1 and cohort 2.

The purpose of this study was to assess the feasibility of resting seated and standing limb occlusion pressure (LOP) measurement with the Delfi Personalized Tourniquet System, evaluate the effectiveness of visual cueing in balancing weight distribution on force plates during LOP assessments, and examine whether acclimation to weight balance distribution without visual cues can maintain consistent weight balance and influence LOP measurements. The experimental trials involved two independent cohorts. Cohort 1 (*n* = 11, 6 female) and Cohort 2 (*n* = 11, 7 female) each underwent three non-randomized conditions with three measurement attempts each on two occasions separated by 48 h (Figure 1) (total of 33 measurement attempts per condition per day). Both cohorts consisted of entirely separate participants, with no overlap between groups. LOP of the dominant leg, classified as the leg used to kick a ball, and weight distribution on the force plate (expressed as a percentage) were recorded at the end of each condition (Figure 2). Participants rested in a seated position for 5 min between trials and were permitted to speak and hydrate with water during these periods. If an LOP determination was unsuccessful, the attempt was recorded as such, and the participant rested for five minutes before proceeding to the next scheduled attempt. No additional attempts were made beyond the three per condition per session. These modified upright positions (leaning, seated, toe-touch) were selected as pragmatic attempts to approximate upright exercise stances while reducing postural variability that interferes with automated LOP detection.

Cohort 1 completed three non-cueing, three visual-cueing, and three acclimation conditions, with five-minute rest intervals between each. In the non-cueing condition, participants stood naturally on the force plates without visual feedback before LOP assessment. In the visual-cueing condition, participants stood while maintaining equal weight distribution between the right and left leg, guided by real-time feedback from the force plate monitor, prior to attempting LOP acquisition. The acclimation condition replicated the visual-cueing protocol but removed visual feedback to assess whether participants could maintain balanced weight distribution independently.

Cohort 2 completed three seated, three leaning, and three toe-touch conditions, with five-minute rest intervals between each. In the seated condition, participants sat on the edge of a chair with back support during LOP assessment, ensuring the cuff was not in contact with the chair. For the leaning condition, participants stood on a force plate and leaned against a wall while maintaining a 40% front and 60% back weight distribution. In the toe-touch condition, participants placed approximately 80% of their body weight on the non-dominant leg while lightly touching the toe of the dominant leg on the force plate (20% weightbearing on dominant leg). No external assistance for balance was provided in any of the conditions.

### Procedures

Data collection occurred in the Exercise Physiology Research Laboratory on two separate days at the same time of day, 48 h apart. All research sessions took place between 0700 and 1300 h in a temperature-controlled lab maintained at 21–23°C. Participants were instructed not to deviate from their usual exercise and dietary routines throughout the study. Additionally, participants fasted for at least 4 h before the session and refrained from exercise, alcohol, and caffeine for 24 h prior to each session.

### Anthropometrics

Anthropometric data were collected on the first day. Body height and weight were measured using a stadiometer and scale (Detecto 439 Physician Beam Scale) while participants stood upright and barefoot. Duplicate dominant and non-dominant thigh circumference measurements were taken while the participant rested supine. A Gulick measuring tape measured the thigh circumference at 33% of the distance from the middle of the inguinal crease to the top of the patella, consistent with previous investigations (11).

### LOP device

LOP was assessed using Delfi's Personalized Tourniquet System (PTS) for Blood Flow Restriction (Delfi, Vancouver, Canada; 11.5 cm cuff width) (Delfi PTS BFR System 9-2200-001BFR: SN:2020D-04294). The cuff was positioned at the highest point of the dominant leg while participants stood. LOP was established by positioning the participant in the experimental condition and pressing the “PTP” button on the Delfi PTS, which automatically determines LOP without the need for further user input. The screen presents LOP as well as customizable options to program at a percentage of LOP for training purposes. We referenced the LOP number for our study. The cuff remained deflated during rest intervals. Given the study's design, participants were aware of the treatment conditions. Cuffs were secured to each participant's dominant limb tightly enough to allow placement of one finger underneath, while remaining firm enough to resist sliding down under the effects of gravity. Cuffs were applied to the limbs with a protective sleeve underneath to minimize friction and maximize likelihood of achieving a successful measurement.

### Force plate

BTrackS^TM^ Balance Plate (Balance Tracking Systems, San Diego, CA) recorded weight distribution during the conditions. Calibration and foot placement conformed to manufacturer specifications.

### Statistical analysis

The determination of LOP was not possible for all participants. Therefore, to evaluate differences in the frequency of participants with successfully identified LOP across the three conditions (categorical comparisons), Cochran's *Q* test for related samples was applied. All statistical analyses were performed using SPSS software (version 24), with statistical significance set at *p* < 0.05.

## Results

Three participants were lost due to scheduling conflicts prior to data collection, leaving 11 participants per cohort. (Table 1). We were unable to obtain all LOP measurements from a single participant in either cohort across all three conditions. A total of 66 attempts were performed in each position (6 per participant) per cohort, with three conditions assessed per cohort. Considering both cohorts, the highest percentage of successful LOP measurements for any participant was 11 out of 18 attempts (61%) and the lowest was 2 out of 18 (11%) attempts. Weight distributions on the BTrackS^TM^ Balance Plate for the different conditions in Cohort 1 and Cohort 2 are displayed on Table 2 and Table 3, respectively.

**Table 1:** Participant baseline characteristics.

Variable	Cohort 1	Cohort 2
Age, yr	19.6 ± 1.2	21.5 ± 4.9
Height, cm	163.2 ± 14.6	170.9 ± 13.6
Weight, kg	67.6 ± 14.7	72.1 ± 20.2
BMI, kg/m^2^	25.6 ± 5.8	24.2 ± 3.7
Seated SBP, mmHg	122 ± 8	119 ± 10
Seated DBP, mmHg	71 ± 4	75 ± 6
Dominant limb circumference, cm	59.0 ± 4.4	58.7 ± 6.5
Non-dominant limb circumference, cm	58.9 ± 4.1	59.3 ± 6.2

All data is presented in mean ± SD. BMI, body mass index; SBP, systolic blood pressure; DBP, diastolic blood pressure.

**Table 2:** Dominant leg weight distribution percentages in cohort 1.

Cohort 1	Session 1			Session 2		
Conditions	Trial 1	Trial 2	Trial 3	Trial 1	Trial 2	Trial 3
Condition 1 (non-cueing)	49.8 ± 2.5	50 ± 1.6	50.7 ± 1.8	49.1 ± 2.4	48.9 ± 2.1	49.7 ± 2.6
Condition 2 (cueing)	50.3 ± 0.6	50.1 ± 0.5	50 ± 0.4	50.1 ± 0.5	50 ± 0.4	50.1 ± 0.3
Condition 3 (acclimation)	50.0 ± 1.5	50.6 ± 1.9	49.5 ± 2.0	50.6 ± 1.3	49.8 ± 1.4	50.6 ± 1.9

The percent weight distribution of dominant leg data is reported as mean ± SD.

**Table 3:** Dominant leg weight distribution percentages in cohort 2.

Cohort 2	Session 1			Session 2		
Conditions	Trial 1	Trial 2	Trial 3	Trial 1	Trial 2	Trial 3
Condition 1 (seated)	41.1 ± 1.6	40.5 ± 0.5	40.8 ± 1.1	41.9 ± 2.2	40.5 ± 1.8	41.4 ± 1.9
Condition 2 (leaning)	58.9 ± 1.6	59.5 ± 0.5	59.2 ± 1.1	58.1 ± 2.2	59.5 ± 1.8	58.6 ± 1.9
Condition 3 (toe-touching)	20.1 ± 2.3	19.6 ± 2.3	18.9 ± 3.3	19.7 ± 2.6	19.4 ± 2.2	19.5 ± 1.3

The percentage weight distribution of the dominant leg data is reported as mean ± SD.

**Figure 3:**
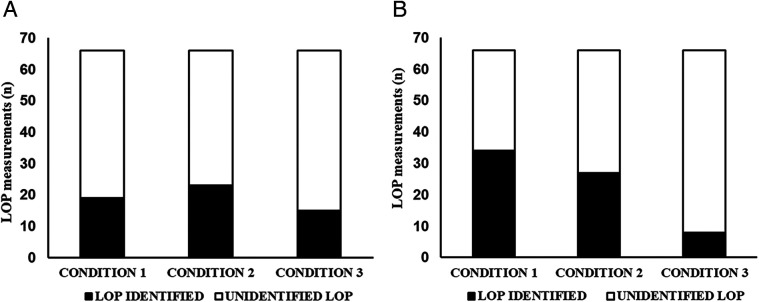
**(A)** Number of LOP measurements (*n*) from cohort 1 after two sessions. Condition 1: LOP standing with 50% weight-bearing; Condition 2: visual feedback on 50%; Condition 3: no feedback after visual feedback condition (acclimation). **(B)** Number of LOP measurements (*n*) from Cohort 2 after two sessions. Condition 1 seated; Condition 2 - leaning against wall with visual feedback on 40/60%; Condition 3 – dominant leg toe touch with 20/80%.

**Figure 4:**
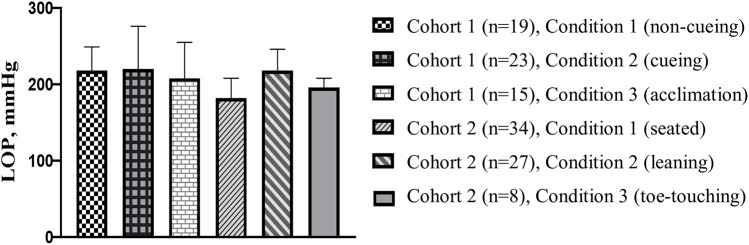
Average LOP for each condition. Each condition comprised a total of 66 LOP measurements from both days. The reported number (*n*) is the number of successful measurements from each condition.

In cohort 1, no significant differences were observed in the frequency of successful LOP determinations across the three tested conditions (Figure 3A). LOP was identified in 19, 23, and 15 assessments under conditions 1, 2, and 3, respectively (Cochran's *Q* = 2.909; *p* = 0.234). For successful attempts, LOP values ranged from 195.8–220.0 ± 12.0–55.8 mmHg across cohorts and conditions (Figure 4).

In cohort 2, successful LOP identification occurred in 34, 27, and 8 assessments under conditions 1, 2, and 3, respectively (Figure 3B). The differences in successful LOP determination were statistically significant between conditions 1 and 3 (*p* < 0.001) and between conditions 2 and 3 (*p* = 0.001).

## Discussion

Based on our pilot data, assessing resting seated or standing LOP using the Delfi PTS was not feasible in most participants, whether under 50% weightbearing or wall-supported (∼80%) conditions. Seated LOP assessment fared better than standing conditions, achieving a successfully measured LOP 51% of the time (*n* = 66 measurement attempts, 34 successful LOPs). Despite attempts to standardize loading, with and without visual feedback, consistent and reliable LOP measurements in standing could not be achieved. These findings indicate that maintaining a stable and reproducible limb loading posture during standing LOP assessment presents substantial practical challenges. Accordingly, our hypothesis was partially supported, as standing LOP proved largely unfeasible in both cohorts; however, seated resting LOP achieved more than 50% success. These successful measurement rates do not fully support its implementation in practice, as the high rates of non-measured LOP could pose a hindrance to practitioners looking to assess LOP in positions outside of supine. Given the limited number of successful determinations across conditions, formal reliability analyses (e.g., ICCs, CVs) were not possible. Accordingly, the most relevant outcome metric for this pilot study was the rate of successful LOP detection across conditions.

Importantly, these results pertain specifically to the Delfi PTS, as its automated measurement algorithm may be particularly sensitive to upright postural variability. In addition, because the Delfi PTS has been validated previously (6) and can apply pressures up to 350 mmHg – sufficient for most limbs – we speculate that the challenges observed are due to the position of assessment and the sensitivity of the device to postural variability, rather than any mechanical limitation of the system itself (Personalized BFR, 2021). Therefore, our findings do not support the use of seated or standing LOP assessment with the Delfi PTS prior to BFR exercise and underscore the need for alternative positioning strategies or device modifications to better account for upright postural variability during LOP determination. Importantly, our study did not aim to establish the reliability of absolute LOP values, but rather to evaluate the feasibility of obtaining a measurable LOP in seated and standing positions. Because successful determinations were achieved in only a subset of participants, formal reliability analyses (e.g., ICCs or coefficients of variation) were not possible. Therefore, the most relevant outcome metric in this pilot study was the rate of successful LOP detection across conditions and differences between conditions.

In Cohort 1, where participants were instructed to maintain 50% weightbearing while standing, successful LOP determination using the Delfi PTS was achieved in only 19 of 66 attempts (28.7%) during the baseline condition. The introduction of visual feedback provided a slight improvement, with 23 of 66 (34.8%) attempts achieving successful measurement, but this effect was not sustained once visual feedback was removed, dropping to 15 of 66 measurements (22.7%). In Cohort 2, seated assessments yielded higher success rates, with 34 of 66 (51.5%) successfully measured. However, the leaning condition, which provided approximately 20% weight support in the medial-lateral direction, resulted in successful measurement in only 27 of 66 (40.9%) measurements. The toe-touch condition, designed to reduce loading on the dominant limb by 80%, produced the worst overall LOP measurement success rates of 8 of 66 (12.1%). Collectively, these results demonstrate substantial variability and overall low feasibility of reliably capturing seated or standing LOP with the Delfi PTS, even when external feedback and partial weight support were implemented.

The inconsistency across conditions and cohorts suggests that LOP assessment is highly sensitive to minor shifts in posture and weight distribution when using the Delfi PTS. The modest gains from visual feedback were not sustained when feedback was removed, indicating that participants were unable to internalize or replicate the targeted loading pattern without ongoing guidance. This lack of reproducibility poses a concern for both research and clinical applications of BFR where standing exercises are performed with the Delfi PTS. Additionally, a nonsignificant trend was observed for successful LOP measurements in leaner participants. It remains unclear whether the amount of subcutaneous fat under the cuff directly affects LOP acquisition when standing, which warrants further investigation. Collectively, these findings argue against the use of seated or standing LOP assessment as a foundation for BFR prescription when using the Delfi PTS and support a shift toward supine LOP testing, which offer more controlled conditions for reliable pressure standardization.

Only one prior study has investigated the reliability of standing LOP assessment using the Delfi PTS in the lower body (3). They reported excellent seated and standing test–retest reliability across two measurements, with an intraclass correlation coefficient of 0.953 [95% CI (0.822–0.988)] and a coefficient of variation of 2.97% for standing, and 0.975 [95% CI (0.932–0.994)] with a coefficient of variation of 1.82% for seated. However, their protocol differed from ours in three keyways. First, participants in the Hughes et al. (3) study stood in the anatomical position for five minutes prior to LOP assessment. In contrast, our protocol attempted to replicate real-world conditions by having participants remain seated during the five-minute rest period, followed by immediate standing and LOP measurement. This difference may account for the limited feasibility of LOP assessment observed in both cohorts of our trial. Second, Hughes et al. (3) did not report how participants distributed their weight during standing LOP measurement. If participants unconsciously offloaded the assessed limb or adopted compensatory strategies, this could have affected the reliability of the measurement, particularly as the Delfi PTS is sensitive to changes such as muscular contraction in the underlying limb. Since these details were not provided, replication of their findings was not possible within the framework of our study. If the device senses any changes while assessing the limb's LOP, the device will be unable to determine LOP, which is what we speculate occurred in all the trials where LOP was not able to be determined. Third, Hughes et al. (3) performed one assessment per position per participant (50 total measurements per position), with 10 individuals completing an additional session to assess reliability. In contrast, our study included fewer participants (*n* = 11) but incorporated multiple assessments per participant (6 each), resulting in a greater total number of measurements per position (*n* = 66). Despite these design differences, participant thigh circumferences were comparable across studies {[Hughes et al. (3): 53–56 cm ± 5–7 cm; Current study Cohorts 1 and 2: 58.7–59.0 cm ± 4.4–6.5 cm]} as were successfully measured LOP values {[Hughes et al. (3) standing: 241.5 ± 49.3 mmHg; Current study Cohorts 1 and 2 standing range: 195.8–220.0 mmHg ± 12.0–55.8 mmHg]}. We speculate that the differences observed between studies are less attributable to thigh circumference and more likely due to methodological factors (e.g., measurement approach, postural nuances, or sample variation). Unlike Hughes et al. (3), who reported high test-retest reliability of seated and standing LOP, our dataset did not provide sufficient successful trials to allow calculation of variability metrics. This difference highlights that while seated and standing LOP may be reliable when it can be obtained, the key limitation in practice is whether LOP can be successfully measured at all.

Our study is not without many limitations. First, we utilized the Delfi PTS, which is highly sensitive to changes beneath the cuff and may produce device errors if any variance is detected during LOP assessment. It remains unclear whether similar challenges would occur with other automated or manually operated cuff systems. Future research could include paired doppler or manual assessments to provide variability data when automatic LOP measures are successful as well as comparing other automatic BFR cuffs. Second, participants rested in a seated position prior to LOP measurement, which may have subtly influenced hemodynamic responses and contributed to the difficulties in establishing LOP in standing conditions. This methodological choice contrasts with that of Hughes et al. (3), who demonstrated high reliability using a five-minute standing rest period prior to assessment. However, our protocol was designed to reflect more realistic, field-based conditions. Future research should examine whether LOP assessments performed in supine positions can be reliably applied to standing or upright BFR exercise, or whether real-time, dynamic LOP calibration is required for such modalities. Third, we did not include a supine LOP assessment, which limits our ability to determine the accuracy of the Delfi PTS in the most regressive exercise position. However, as the Delfi PTS has been previously validated in supine (6), we did not consider it necessary as a comparator in this trial. Future studies may incorporate a supine LOP assessment to further support data interpretation. Fourth, we did not randomize participants to each condition. However, this was by design as we wanted to evaluate the impact of visual cueing on LOP feasibility in standing. Fifth, the adults included within this analysis are healthy and free of injury. Extrapolation to clinical practice warrants caution. Lastly, this was a pilot study. While our findings suggest limited feasibility of seated and standing LOP assessment, the small sample size limits generalizability. However, we included 6 measurements per participant (contrasting with prior studies) along with stringent postural weightbearing conditions guided by force plate technology prior to assessment of LOP. It remains unknown whether a larger cohort or different postural cueing conditions would yield different results.

The results of this pilot study do not support the feasibility of determining LOP in the standing position using the Delfi PTS, as the procedure proved unsuccessful in most participants—regardless of whether equal weightbearing was cued or supported using a wall. We also had limited success with seated LOP assessment (51% success rate). These findings raise concerns about the reliability of seated and standing LOP assessments and underscore the need for further research to identify strategies, cues, or methodological adjustments that may reduce error rates and improve measurement reliability in upright positions.

## Data Availability

The raw data supporting the conclusions of this article will be made available by the authors, without undue reservation.
